# Form and function of damselfish skulls: rapid and repeated evolution into a limited number of trophic niches

**DOI:** 10.1186/1471-2148-9-24

**Published:** 2009-01-30

**Authors:** W James Cooper, Mark W Westneat

**Affiliations:** 1 Department of Organismal Biology & Anatomy, University of Chicago, Chicago, IL 60637, USA; 2 Department of Zoology, Field Museum of Natural History, Chicago, IL 60605, USA; 3 Department of Biology, Syracuse University, Syracuse, NY 13244, USA

## Abstract

**Background:**

Damselfishes (Perciformes, Pomacentridae) are a major component of coral reef communities, and the functional diversity of their trophic anatomy is an important constituent of the ecological morphology of these systems. Using shape analyses, biomechanical modelling, and phylogenetically based comparative methods, we examined the anatomy of damselfish feeding among all genera and trophic groups. Coordinate based shape analyses of anatomical landmarks were used to describe patterns of morphological diversity and determine positions of functional groups in a skull morphospace. These landmarks define the lever and linkage structures of the damselfish feeding system, and biomechanical analyses of this data were performed using the software program JawsModel4 in order to calculate the simple mechanical advantage (MA) employed by different skull elements during feeding, and to compute kinematic transmission coefficients (KT) that describe the efficiency with which angular motion is transferred through the complex linkages of damselfish skulls.

**Results:**

Our results indicate that pomacentrid planktivores are significantly different from other damselfishes, that biting MA values and protrusion KT ratios are correlated with pomacentrid trophic groups more tightly than KT scores associated with maxillary rotation and gape angle, and that the MAs employed by their three biting muscles have evolved independently. Most of the biomechanical parameters examined have experienced low levels of phylogenetic constraint, which suggests that they have evolved quickly.

**Conclusion:**

Joint morphological and biomechanical analyses of the same anatomical data provided two reciprocally illuminating arrays of information. Both analyses showed that the evolution of planktivory has involved important changes in pomacentrid functional morphology, and that the mechanics of upper jaw kinesis have been of great importance to the evolution of damselfish feeding. Our data support a tight and biomechanically defined link between structure and the functional ecology of fish skulls, and indicate that certain mechanisms for transmitting motion through their jaw linkages may require particular anatomical configurations, a conclusion that contravenes the concept of "many-to-one mapping" for fish jaw mechanics. Damselfish trophic evolution is characterized by rapid and repeated shifts between a small number of eco-morphological states, an evolutionary pattern that we describe as reticulate adaptive radiation.

## Background

Studies of functional morphology permit us to describe the connection between anatomical and ecological diversity in terms of biomechanical ability [1,2]. By examining the trophic anatomy of a lineage using both morphometric and biomechanical analyses, we can improve our understanding of how the diversification of head morphology is linked to ecological divergence via changes in feeding performance [3]. Further details about patterns of functional diversification can be obtained by performing these studies within a comparative, phylogenetic context [4,5]. Such an approach can be used to indicate which aspects of trophic biomechanics tend to be correlated, whether they have evolved rapidly or exhibit evidence of constraint, and whether there are significant biomechanical differences between the related members of separate trophic groups.

The damselfishes represent one of the most successful radiations of coral reef fishes [6-9], containing nearly 300 species that are associated with coral communities [10]. Pomacentrids are ubiquitous on coral reefs [6], and they have been present within these ecosystems for at least 50 million years [9,11]. Damselfishes are also widely distributed among the nearshore rocky reef communities of both tropical and temperate regions, although they play less dominant roles in these ecosystems [10]. By studying the functional morphology of the Pomacentridae we can describe the radiation of a successful vertebrate lineage in both anatomical and mechanical terms [12,13], and such work has strong implications for understanding the evolutionary history of important ocean ecosystems, particularly coral reefs.

Here we describe damselfish head shape diversity in a manner that explicitly links morphology to ecology via biomechanics. The field of functional morphology has seen a recent increase in the number of studies that combine shape analyses with biomechanical analyses [e.g., [4,5,14-20]], and some of these authors have utilized the advantages that geometric, or coordinate based, morphometric techniques provide when engaging in this work [e.g., [15,16,18,19]]. Coordinate based shape analyses require fewer assumptions regarding the relative importance of individual variables than do traditional (measurement based) shape analyses, they facilitate the detection of morphological patterns that are not specified *a priori*, and they permit the reliable removal of size and orientation differences from an anatomical dataset [15,16,18,19,21,22].

We used a combination of geometric morphometrics, biomechanical computer models, and phylogenetic comparative techniques to examine patterns of trophic diversity among the Pomacentridae. The goals of this study were to: (1) perform dissections on the heads of specimens representing all damselfish genera and trophic classes, (2) collect coordinate data for anatomical landmarks of functional importance to damselfish feeding from digital images of these dissections, (3) use this data to describe and quantify the shape diversity of damselfish heads using geometric morphometric techniques, (4) use a computer model to predict the biomechanical potential of damselfish skulls based on the relative positions of these landmarks, and (5) use phylogenetic comparative techniques to examine patterns in the evolution of damselfish trophic biomechanics.

## Methods

Damselfish specimens were primarily selected from the fish collection of the Field Museum of Natural History, and additional specimens were provided by the Australian Museum, the Scripps Institution of Oceanography and the United States National Museum of Natural History in the Smithsonian Institution. At least one species from each of the 28 damselfish genera (until recently 29) was examined [10,23,24], and in almost all cases we examined 3 specimens of each species (Additional file 1). Most of the specimens were collected by the authors and their collaborators during Field Museum expeditions to the Philippines, Australia and Cape Verde. Fishes were killed using spears and the ichthyocide rotenone in accordance with the permits issued by the governmental agencies of these countries.

**Figure 1:**
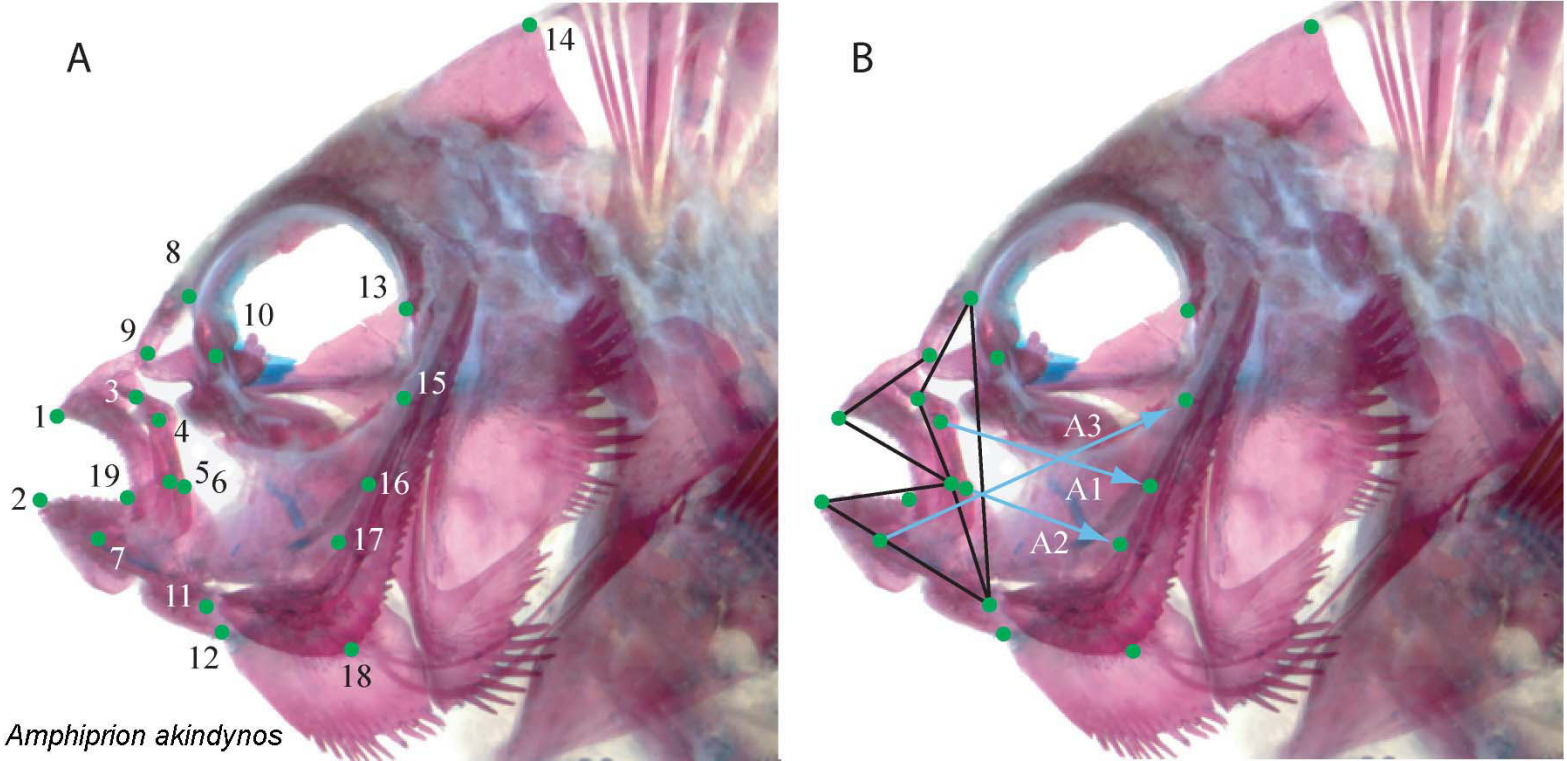
**Anatomical landmarks and biomechanical linkages of functional importance for damselfish feeding**. A. Landmarks used in morphological and biomechanical analyses: 1 = Tip of the anterior-most tooth on the premaxilla; 2 = Tip of the anterior-most tooth on the dentary; 3 = Maxillary-palatine joint (upper rotation point of the maxilla); 4 = Insertion of the A1 division of the *adductor mandibulae *on the maxilla; 5 = Maxillary-articular joint (lower point of rotation of the maxilla); 6 = Insertion of the A2 division of the *adductor mandibulae *on the articular process; 7 = Insertion of the A3 division of the *adductor mandibulae *on the anterior, medial surface of the articular; 8 = Posterior tip of the ascending process of the premaxilla; 9 = Joint between the nasal bone and the neurocranium; 10 = The most anterio-ventral point of the eye socket; 11 = Articular-quadrate joint (lower jaw joint); 12 = Insertion of the interopercular ligament on the articular (point at which moth opening forces are applied); 13 = Most posterio-ventral point of the eye socket; 14 = Dorsal-most tip of the supraoccipital crest on the neurocranium; 15 = Most dorsal point on the origin of the A3 division of the adductor mandibulae on the preopercular; 16 = Most dorsal point on the origin of the A1 division of the *adductor mandibulae *on the preopercular; 17 = Most dorsal point on the origin of the A2 division of the *adductor mandibulae *on the preopercular; 18 = Posterio-ventral corner of the preopercular; 19 = Corner of the mouth. B. Levers and linkages in damselfish skulls, with schematics of the three divisions of the *adductor mandibulae*.

Dissections were performed on the right side of fish heads in order to expose morphological landmarks of functional importance for fish feeding (Figure 1). Except in the case of *Altrichthys curatus*, where only juveniles were available, all specimens were adults. Dissected heads were photographed in lateral view from a position directly perpendicular to the plane described by the landmarks on the side of each fish's head. Since identical positioning of moveable elements is necessary when performing geometric morphometric analyses, all fishes were photographed with their mouths closed and their operculae and hyoid arches adducted. A scale bar was included in each photograph. Most specimens were photographed using a Leica digital camera interfaced with a dissecting microscope. Larger species were photographed using a Nikon coolpix 4300 digital camera. Nineteen morphological landmarks (LM; Figure 1) were plotted on each image using the software program tpsDig [25], which was also used to determine the Cartesian coordinates of each landmark and to establish the scale of all images.

### Shape analyses

The coordinate data generated using tpsDig [25] were used to determine the mean skull shape for each species. GLS Procrustes superimpositions of the coordinate data from all specimens of each species were performed using the program CoordGen (species were analysed individually). This procedure transforms the data so as to reduce or eliminate differences in landmark configurations that are due to the size of the specimens or their positions when photographed, and does so without distorting shape information. Landmark configurations are translated, scaled and rotated so as to minimize the squared, summed distances between the corresponding landmarks of each individual and the mean [26]. The average shape of each species was determined by calculating the mean of each coordinate from the Procrustes transformed data from all specimens of that species using the program Excel (Microsoft, Corp.).

One-way MANOVA analyses were conducted in order to determine it there were significant skull shape differences between damselfish trophic groups. Analyses were performed using the Procrustes mean shape of each species, with every species except *Cheiloprion labiatus *assigned to one of three trophic groups (Additional file 1). *C. labiatus *was excluded from these analyses since it was the only member of its trophic category. Two different MANOVA test statistics were used to determine if there was trophic information in the morphological data: Wilk's Lambda (using the program CVAGen6o), and Goodall's F (using the program MANOVAboard). Pairwise one-way MANOVA analyses (Goodall's F test) were performed using the program Twogroup6h in order to determine if pairs of trophic groups (including a planktivore vs. omnivore + herbivore comparison) possess significantly different skull shapes. CoordGen, CVAGen6o, MANOVAboard, and Twogroup6h are part of the Integrated Morphometrics Programs (IMP), and compiled stand-alone versions that run in Windows are freely available at .

A canonical variates (CV) analysis of the Procrustes means of each species (except *C. labiatus*) was also performed using the program CVAGen6o. The results of this analysis were used to determine how many types of independent shape variation (CV axes) were present in the data, to depict patterns of damselfish skull shape variation, and to assess the reliability with which CV axes could be used to assign a species to the appropriate trophic group based on its mean Procrustes coordinate configuration.

Partial warp scores were calculated for the specimens (CVAGen6o), and these scores were used to find the set of axes that allows for the greatest possible ability to discriminate between two or more groups. The number of distinct CV axes were determined at a p = 0.05 level of significance, and the canonical variates scores of all the specimens were computed. We used these scores to plot the location of each species on CV axes in order to depict shape differences among the damselfish species in the three trophic groups examined. A jackknife resampling analysis (1,000 iterations) was also performed using CVAGen6o in order to determine how reliably the CV axes could be used to assign specimens to trophic groups. This procedure leaves out one specimen (in this case the Procrustes mean for a species) at a time and then assigns that species to a group based on the CV axes.

A relative warp (RW) analysis was also used to depict head shape variation among the specimens. The software program tpsRelw [27] was used to perform a Procrustes sumperimposition of the coordinate data (all specimens) generated using tpsDig [25], followed by a RW analysis of the transformed coordinates. The RW scores of each specimen were used to map their location on RW axes, and 2-dimensional views of this "shape space" were inspected in order to determine the relative shape differences among individual skulls. We chose to examine damselfish head shape distributions along those initial RW axes that, when taken together, summarized at least 70% of the variation in the data

### Biomechanical analyses

The mechanical properties of damselfish jaw linkages, as well as predictions of their kinetic potential, were modelled using the computer program JawsModel4 (available from Mark Westneat upon request). The multiple mobile bones in most perciform fish skulls form a connected series of levers and linkages whose properties can be described in mechanical terms [28-32]. JawsModel4 uses engineering principals to describe and predict the biomechanics of the anterior jaw linkages during fish feeding [30]. The anatomical linkages in damselfish skulls that were modelled using JawsModel4 can be seen in Figure 1. Only data from adult specimens were used as input, so there are no calculations for *Altrichthys curatus*.

JawsModel4 was used to calculate two sets of mechanical parameters for particular sub-divisions of damselfish trophic anatomy: 1) simple mechanical advantage (MA); and 2) kinematic transmission coefficients (KT). MA describes the length ratio of two levers connected to the same fulcrum: an input lever (inlever), or effort arm, and an output lever (outlever), or resistance arm (MA = input lever length/output lever length).

In most fishes the jaws are closed by the *adductor mandibulae *(*AM*) muscle, and in many perciform fishes, including the damselfishes, this muscle is sub-divided into three parts (the A1, A2, A3) that insert at different locations on the lower jaw (Figure 1). The distances between the insertion points of the 3 *AM *sub-divisions and the center of the lower jaw joint (the quadrate-articular joint) form three inlevers, while the distance from this joint to the tip of the anterior-most tooth in the lower jaw was defined as the outlever. High mechanical advantages (relatively long inlevers) allow for a multiplication of the force produced by the jaw muscles during its transfer to the lower jaw and the teeth. Low mechanical advantages (relatively long outlevers) allow for a faster bite, as a relatively small retraction of the input lever is translated into a much wider sweep of the lower jaw in the same amount of time.

Kinematic transmissions coefficients (KTs) describe the ratio of output rotation to input rotation (KT = output rotation/input rotation) when motion is transmitted through a set of connected linkages [30]. JawsModel4 calculates the transmission of rotational motion through independent four-bar linkages, or through a series of connected four-bars, and calculates KT values for each action. High KT values indicate high levels of velocity transfer through a linkage system, with a low KT signifying greater force transmission. Four mechanical advantage and three KT parameters were estimated for each specimen: the jaw opening MA, the MAs employed during biting by the three biting muscles (A1MA, A2MA, A3MA) and the KTs for the rotation of the maxilla (maxillary KT), the production of gape or mouth opening (gape KT), and the protrusion of the premaxilla (i.e., upper jaw protrusion; protrusion KT), using the protocol originally outlined by Westneat [30].

### Phylogenetics

**Figure 2:**
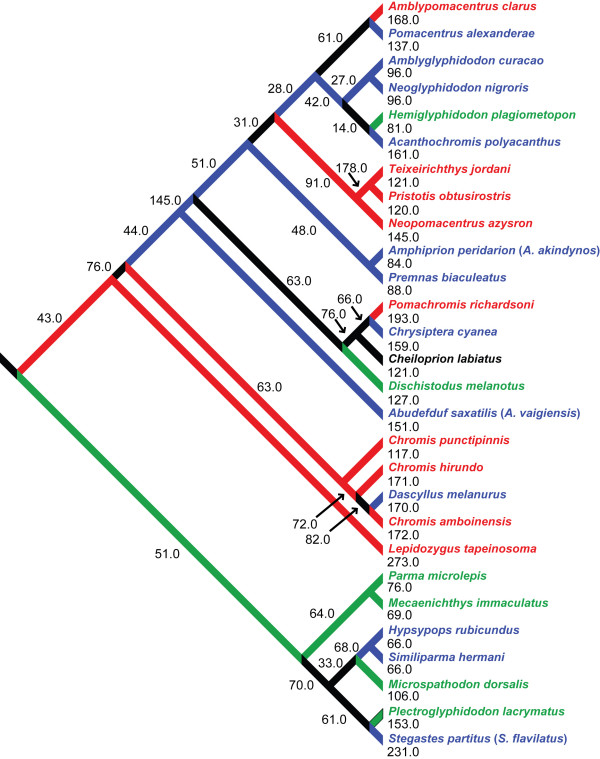
**The damselfish phylogeny used in the phylogenetic comparative analyses in this study**. Numbers indicate branch lengths. This tree is derived from a Bayesian consensus tree computed from nuclear and mitochondrial genetic data from 104 damselfish species and all 29 damselfish genera [68,69]. *Abudefduf vaigiensis*, *Amphiprion peridarion *and *Stegastes partitus *are used to estimate the positions of their cogeners: *Abudefduf saxatilis*, *Amphiprion akindynos *and *Stegastes flavilatus*. Planktivores (red), Herbivores (green), Omnivores (blue)

In order to account for the influence of common descent on the pattern of damselfish biomechanical diversity, it was necessary to perform comparative analyses within a phylogenetic context. We used the topology and branch lengths from a well-supported Bayesian consensus tree in all phylogenetic comparative analyses [24]. Three of the species examined here were not included in the phylogenetic study: *Abudefduf saxatilis*, *Amphiprion akindynos *and *Stegastes flavilatus*. The phylogenetic locations of *Abudefduf vaigiensis, Amphiprion peridarion *and *S. partitus *were therefore substituted for their congeners. The DNA from members of the genera *Nexilosus *(which is monotypic) and *Altrichthys *(two species) were amplified from dried skeletal remains several decades old, and from formalin fixed tissues, respectively. The limited amount of sequence data obtained, while indicating tentative positions for these species within the damselfish tree [24], did not yield adequate branch length information for comparative analyses. The biomechanical data for *Nexilosus latifrons *and was therefore excluded from the comparative analyses along with that of *Altrichthys curatus*. The phylogenetic relationships of the species examined, and their substitutes, are presented in Figure 2.

### Phylogenetic comparative analyses and statistical tests

Phylogenetically independent contrasts (PIC) analyses were performed using the programs PDAP 1.09 [33] and Mesquite 1.12 [34] in order to search for significant correlations between the biomechanical parameters calculated by JawsModel4. This method was used to reduce the effect of common descent on patterns of correlation between biomechanical variables [35]. PIC calculates contrasts between pairs of branches descended from a common ancestor, and these contrasts may be used in regression analyses [35]. The biomechanical parameter values for each species were determined by calculating a mean value for each parameter from all specimens of that species, and these species means were used in all phylogenetic comparative analyses.

Two fundamentally different methods were used to test for the presence of phylogenetic signal (a tendency for related species to resemble each other) among the patterns presented by each biomechanical parameter. The basic assumption in both cases was that slowly evolving characters will be most similar in closely related species (i.e., the data will display a phylogenetic pattern), while rapidly evolving characters will display a more random pattern. Both of the programs used to perform these analyses are Matlab modules (The mathworks, Inc.).

A randomization procedure was performed using the program PHYSIG.M [36] in order to determine whether a given tree (topology and branch lengths) has a better fit to tip data (in this case values for a specific biomechanical parameter for each species) in comparison to when the data have been randomly permuted across the tips of the tree. A significantly greater fit of the tree to the original data configuration is interpreted as evidence for the presence of significant phylogenetic signal, which suggests that the character (e.g., biomechanical parameter) in question has not evolved rapidly [36].

The fit of two types of linear regression models to the biomechanical data (the JawsModel4 predictions) were compared using the program Regressionv2.m [37]: an ordinary least squares (OLS) linear regression model, which assumes a star phylogeny (i.e., equal levels of inter-relatedness among all members of a clade); and a phylogenetic generalized least squares (PGLS) linear regression model that incorporates phylogenetic information via a branch length matrix. Both ln likelihood values (ln ML) and Akaike information criterion values (AIC) were used to compare the fit of the two models to the data. A better fit is indicated by higher ln ML values and lower AIC values. A better fit of the PGLS model is interpreted as an indication of the presence of significant phylogenetic signal for the character being examined [37].

Regressionv2.m can also be used to weight mean character values in order to account for phylogenetic effects. In cases of significant levels of phylogenetic signal, Regressionv2.m was used to perform analyses of variance (ANOVA), which it does using OLS regression, in order to determine whether the adjusted mean parameter values of the three major damselfish trophic groups were significantly different. Data from those parameters that did not exhibit a significant level of phylogenetic signal were analyzed using the computer program SAS (SAS Institute, Inc.) to calculate pairwise ANOVAs in order to test for biomechanical differences between trophic groups. Gabriel's test, the Studentized maximum modulus test, and Bonferroni t-tests were all used to test for significance when making multiple comparisons. A stepwise discriminant function analysis (DFA) of all biomechanical data (untransformed by phylogenetic correction) was also performed using SAS in order to determine which parameters best distinguished between trophic groups. A nominal alpha level of 0.05 was used to determine significance among the results of all statistical tests.

## Results

### Summary

The main results of this study are: (1) MANOVA, CV and RW analyses effectively distinguished between the cranial morphologies of primarily planktivorous damselfishes and those that consume algae (both herbivores and omnivores), while the skull and jaw muscle anatomy of herbivores and omnivores displayed a great deal of overlap; (2) damselfishes that are primarily planktivorous are strongly distinguished from other pomacentrids by their cranial biomenchanics; (3) damselfish cranial biomechanics have evolved under low levels of phylogenetic constraint; (4) the MA values employed by the three biting muscles have not evolved in a correlated manner, while there has been a considerable degree of correlated evolution among the KT values associated with damselfish jaw linkages; and (5) of the biomechanical parameters examined, the MA values and protrusion KT were associated with damselfish trophic groups to a greater degree than the remaining KT parameters.

### Shape analyses

The results of the one-way MANOVA analyses that examined all species indicated the presence of significant trophic signal in the morphological data (Table 1). Pairwise MANOVA results revealed that damselfish planktivores possess head shapes that are significantly different from both herbivores and omnivores, considered both separately and together (Table 1). Members of these two later groups could not be reliably distinguished from each other by their head anatomy (Table 1).

**Table 1:** One-way MANOVA results. The test statistics used in each case are listed.

Analyses of all data		
Test statistic: Wilk's Lambda:	Lambda = 0.2011	chisq = 40.0954
	df = 6	p < 0.0001
Test statistic: Goodall's F:	F = 6.2098	df (num.) = 68
	df (denom.) = 884	p < 0.01

Pairwise comparisons of trophic groups (Goodall's F)		
Omnivores vs. herbivores::	F = 1.21	df (num.) = 34
	df (denom.) = 578	p = 0.19776
Planktivores vs. omnivores	F = 9.03	df (num.) = 34
	df (denom.) = 714	p < 0.01
Planktivores vs. herbivores:	F = 7.75	df (num.) = 34
	df (denom.) = 476	p < 0.01
Planktivores vs. (omnivores + herbivores)	F = 11.65	df (num.) = 34
	df (denom.) = 918	p < 0.01

**Figure 3:**
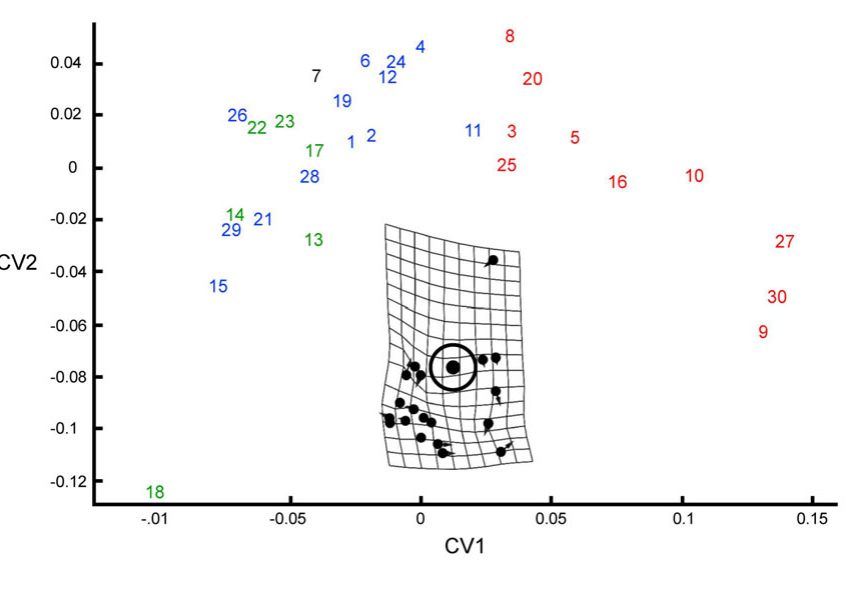
**Canonical variates (CV) score plot for CV axes 1 and 2**. Herbivores in green, Omnivores in blue, planktivores in red, and *Cheiloprion labiatus*, which eats coral polyps, in black. The location for *C. labiatus *was based on the results of relative warps analyses. The number key for individual fishes is: *Abudefduf vagiensis *(1), *Acanthochromis polyacanthus *(2), *Altrichthys curatus *(3), *Amblyglyphidodon curacao *(4), *Amblypomacentrus clarus *(5), *Amphiprion akindynos *(6), *Cheiloprion labiatus *(7), *Chromis amboinensis *(8), *Chromis hirundo *(9), *Chromis punctipinnis *(10), *Chrysiptera cyanea *(11), *Dascyllus melanurus *(12), *Dischistodus melanotus *(13), *Hemiglyphidodon plagiometopon *(14), *Hypsypops rubicundus *(15), *Lepidozygus tapeinosoma *(16), *Mecaenichthys immaculatus *(17), *Microspathodon dorsalis *(18), *Neoglyphidodon nigroris *(19), *Neopomacentrus azysron *(20), *Nexilosus latifrons *(21), *Parma microlepis *(22), *Plectroglyphidodon lacrymatus *(23), *Pomacentrus alexanderae *(24), *Pomachromis richardsoni *(25), *Premnas biaculeatus *(26), *Pristotis obtusirostris *(27), *Similiparma hermani *(28), *Stegastes flavilatus *(29), *Teixeirichthys jordani *(30).

Only the first CV axis was found to significantly distinguish between damselfish trophic groups, and plotting the CV scores of the species examined onto CV axes 1 and 2 revealed that planktivores were strongly distinguished from both herbivores and omnivores by CV 1, but that some omnivores were more similar to planktivores than any of the herbivores (Figure 3). There was strong overlap between the head shapes of herbivores and omnivores (Figure 3). The vector and deformation plot in figure 3 demonstrates that CV1 describes differences in head length, head height, the length of the lower jaw, the length of the ascending process of the premaxilla, the size of the eyes, the size of all jaw adducting muscles, the height of the supraoccipital crest, and the anterior-posterior positioning of the posterior margin of this crest (Figure 3). The use of jackknifing to assign individual species to trophic groups based on the CV axes resulted in the false classification of 3 of the 10 planktivorous species as omnivores, 4 of the 13 omnivores as herbivores, and 3 of the 6 herbivores as omnivores (65.52% accuracy). Planktivores were never classified as herbivores and *vice versa*.

**Figure 4:**
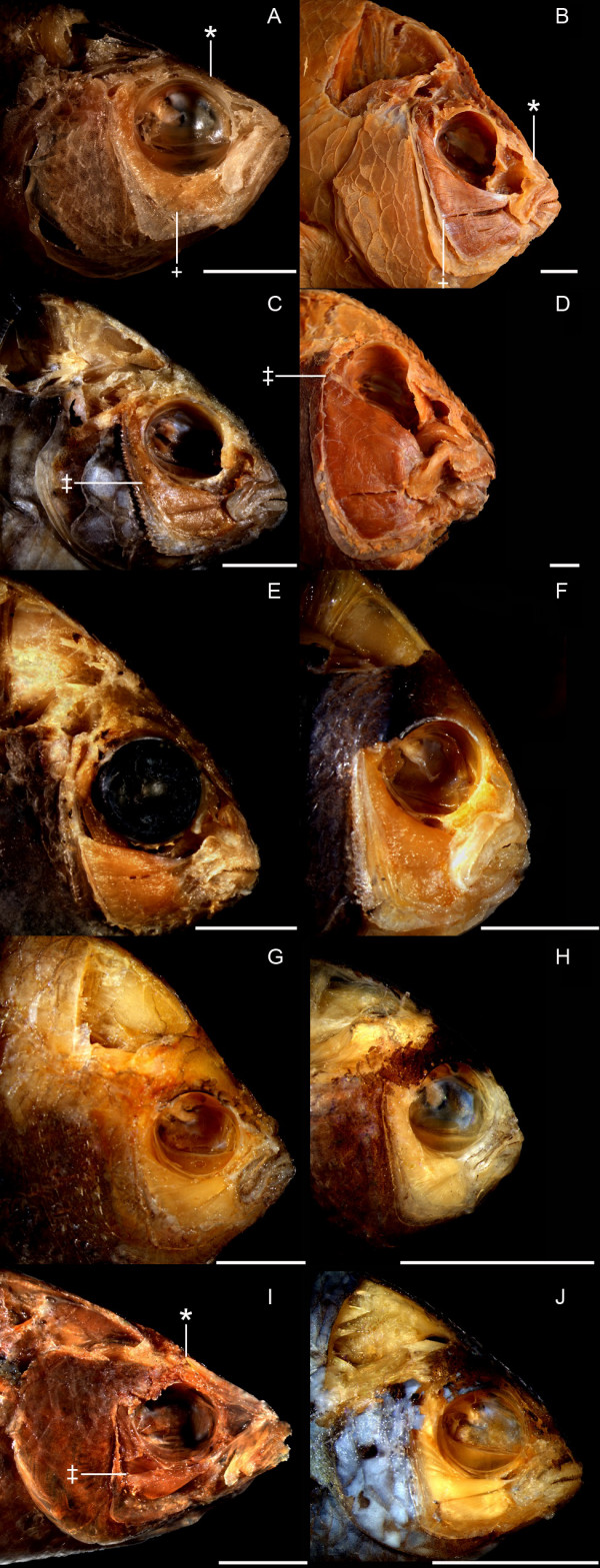
**Pictorial descriptions of relative warps axes and the morphological extremes in each damselfish trophic class**. Plates A-H display pairwise comparisons of damselfishes whose head shapes are strongly separated along one RW axis, but which are otherwise very similar (they have similar scores on other axes). Plate A: *Teixeirichthys jordani *(RW1-). Plate B: *Hypsypops rubicundus *(RW1+). Plate C: *Dischistodus melanotus *(RW2-). Plate D: *Microspathodon dorsalis *(RW2+). Plate E: *Plectroglyphidodon lacrymatus *(RW3-). Plate F: *Amphiprion akindynos *(RW3+). Plate G. *Chromis amboinensis *(RW4-). Plate H: *Neopomacentrus cyanomos *(RW4+). Plate I: *Azurina hirundo*. Plate J: *Chrysiptera cyanea*. Plates B and J depict the range of variation in the head shapes of damselfish omnivores. Plates C and D depict the range of variation in the head shapes of damselfish herbivores. Plates G and I depict the range of variation in the head shapes of damselfish planktivores. _* _= the posterio-dorsal tip of the ascending process of the premaxilla. **+ **= the dorsal edge of the origin of the A2 division of the *adductor mandibulae *on the preopercular bone. ‡ = the dorsal edge of the origin of the A1 division of the *adductor mandibulae *on the preopercular bone. All scale bars = 1.0 cm.

The first four RW axes accounted for a combined total of 71.74% of the variance in our coordinate data: RW1 (35.18%), RW2 (18.36%), RW3 (11.01%), RW4 (7.19%). The head shape differences associated with RW1 are nearly identical with those that are associated with CV1 (see above), and the groupings of the individual damselfish specimens from each species on this axis are therefore extremely similar to those found for the Procrustes mean of each species on CV1. The RW scores of specimens from all trophic groups overlap to a large degree on RW 2–4. RW2 describes variation in the vertical positions of the posterio-dorsal edge of the A1 and A3 muscles, as well as the vertical position of the eyes (Figure 4). RW3 describes variation in the angle of the mouth relative to the long axis of the body (Figure 4). It distinguishes between those fishes whose lower jaws are parallel with their main body axis when the mouth is closed, and those fishes with upturned mouths (i.e., fishes whose jaw lengths contribute relatively less to their overall body length). RW4 describes variation in the bluntness of the head profile (Figure 4).

The RW1 vs. RW2 score plot displayed a distribution pattern that was nearly identical to that in the CV1 vs. CV2 score plot, and this RW information was therefore used to estimate the location of *Cheiloprion labiatus *in Figure 3. *C. labiatus *always fell within the herbivore/omnivore clusters on RW 1–4. Due to the redundancy between many of the CV and RW score plots, we do not present this information for the RW results. RW analyses do, however, permit the description of the major types of uncorrelated shape variation in morphological data, and are therefore useful for describing damselfish skull shape diversity. We therefore provide figure 4, which displays pictorial descriptions of the shape differences that are associated with each RW axis using pair-wise comparisons of pomacentrid species whose head shapes are strongly differentiated by one axis, but which are otherwise similar (i.e., they have similar scores on all other RW axes).

### Biomechanics and phylogenetic comparative analyses

Pomacentrid planktivores were, in general, well distinguished from other damselfishes by their jaw mechanics. *Azurina hirundo*, *Chromis punctipinnis*, *Lepidozygus tapeinosoma*, *Pristotis obtusirostris*, and *Teixeirichthys jordani *had especially low A1MA and A2MA values combined with high protrusion KT values (Additional files 2 and 3), which indicates that they can bite quickly and protrude their upper jaws with a high degree of efficiency. These three parameters best distinguished between damselfish tropic groups (Additional files 4 and 5), and these five species also had some of the most distinct head shapes (Figure 3). Pomacentrid herbivores and omnivores, although possessing generally higher A1MA and A2MA values and lower protrusion KT values than most planktivores, did not show as high a degree of association between their scores for these three parameters (Additional files 2, 3, 4).

**Figure 5:**
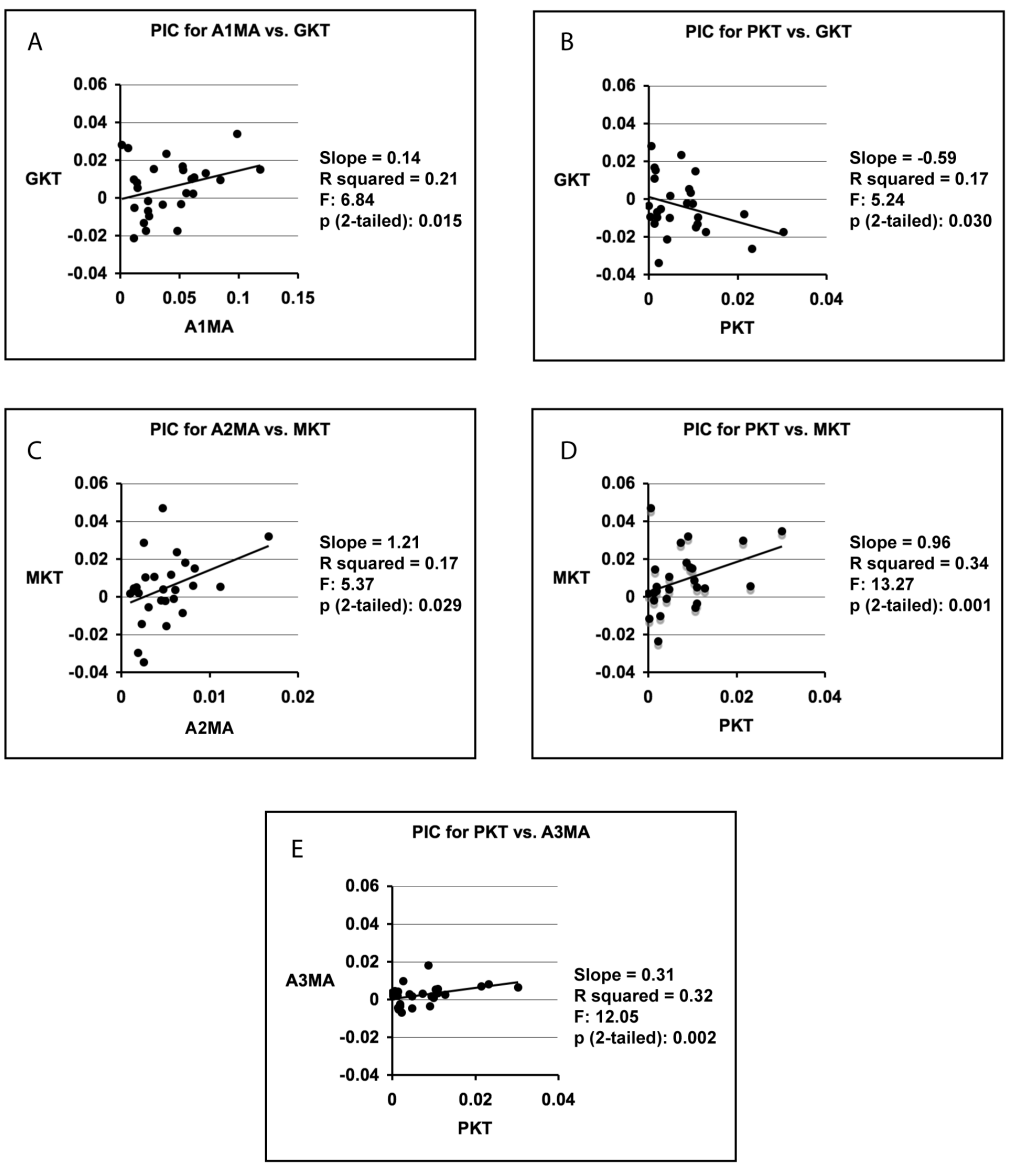
**Regression plots (27 data points) for those phylogenetically independent contrasts (PIC) that were statistically significant**. Maxillary KT = MKT. Gape KT = GKT. Protrusion KT = PKT

The results of the PIC analyses indicate that the evolution of each of the KT parameters examined has been correlated with that of at least two other biomechanical parameters (protrusion KT was significantly correlated with three), while none of the individual MA parameters exhibited evidence of correlated evolution with more than one other parameter, and there was no evidence for correlated evolution between jaw opening MA and any other parameters (Figure 5). There was no evidence for correlated evolution between any of the three biting MA parameters examined (Figure 5).

The PHYSIG results indicated that only one biomechanical parameter, gape KT, showed significantly more phylogenetic signal than would be expected from unconstrained evolution, but the Regressionv2.m results indicated that the fit of the gape KT data to the OLS and PGLS models was similar (Additional file 6). The Regressionv2.m results clearly support the fit of the data from the other six biomechanical parameters to an OLS model (i.e., no significant phylogenetic signal; Additional file 5). The ANOVA results generated by Regressionv2.m for phylogenetically corrected parameter values showed no significant differences between trophic groups for gape KT.

The ANOVA results (Additional file 4) showed significant differences between damselfish trophic groups for three of the four MA parameters (jaw opening MA, A1MA, and A2MA), and one KT parameter (protrusion KT). The results of Gabriel's test, the Studentized maximum modulus test, and Bonferroni t tests all concurred in their indications of which pairwise comparisons of trophic groups were significant for these parameters, and of the seven significant comparisons, six included planktivores (Additional file 4). The results of the stepwise discriminant function analysis indicated that the scores for the biomechanical parameters A1MA, A2MA and protrusion KT, tend to distinguish damselfish trophic groups from each other (Additional file 4).

Planktivorous damselfishes employ low mechanical advantages when using their A1 muscles to close their mouths, and they protrude their upper jaws (premaxillae) with high efficiency (Additional file 4). Omnivores have high MA values associated with their A2 muscles (Additional file 4). Herbivores are never distinguished from both omnivores and planktivores by any biomechanical parameter, but in comparison to planktivores they have higher MA scores for both jaw opening and the A1 muscle, and lower scores for the efficiency with which they protrude their premaxillae (Additional file 4). When compared to omnivores, they employ lower MA values when contracting the A2 (Additional file 4).

Some species have very different MA rankings for different biting muscles (Additional file 2). The planktivores *Azurina hirundo*, *Teixeirichthys jordani *and *Lepidozygus tapeinosoma *have low MA scores for the A1 and A2 muscles, while possessing high scores for the A3 (Additional file 2). *A. hirundo *has the highest A3MA score and the lowest A1MA score (Additional file 2). A different pattern is exhibited by the omnivores *Abudefduf saxatilis *and *Similiparma hermani*, which have the highest A1MA and A2MA scores, respectively, while possessing low A3MA scores (Additional file 2).

Although the minimum MA scores calculated for the three biting muscles were similar, their ranges and maximum values were very different. Approximately 80% of the species examined had A1MA values that were higher than both the maximum A2MA and A3MA values, and the maximum A1MA value was 4.6 times higher than the maximum A2MA score, and 6.1 times higher than the maximum A3MA score (Additional file 2). The single coral eating damselfish species examined, *Cheiloprion labiatus*, has high jaw opening MA and A2MA scores, but is not otherwise greatly distinguished from other damselfishes by its jaw biomechanics.

Two species have very different KT rankings for the rotation of different jaw linkage elements (Additional file 3). *Teixeirichthys jordani *has the lowest gape KT score, the highest protrusion KT score, and the second highest maxillary KT score (Additional file 3). *Chromis punctipinnis *has the second lowest maxillary KT score (along with *Mecaenichthys immaculatus*), the second lowest gape KT score (along with *Similiparma hermani*), and a high protrusion KT score (Additional file 3). *Microspathodon dorsalis *exhibits a pattern of consistently low KT values, and has the lowest maxillary KT score, the lowest protrusion KT score, and along with *Amblypomacentrus clarus*, it has the third lowest gape KT score (which is only marginally different from the second lowest score; Additional file 3).

## Discussion

The results of both the morphological and biomechanical analyses clearly indicate that the evolution of planktivory has involved important changes in pomacentrid functional morphology. The shape differences that separate planktivores and non-planktivores account for the greatest degree of damselfish skull shape variation (Figure 3), and of those biomechanical parameters that most clearly distinguish between trophic groups, planktivores exhibit different values compared to non-planktivores in most cases (Additional files 2, 3, 4, 5). These aspects of damselfish trophic biomechanics have also experienced relatively high rates of evolution (i.e., they have experienced low levels of phylogenetic constraint; Additional file 6).

Damselfish planktivory can be described as particulate feeding in that they attack individual plankters that they select visually [38-40]. The possession of relatively large eyes should increase these fishes' ability to find and target planktonic prey (Figure 4), and the mechanical arrangements of their head anatomies should allow them to rapidly pick plankton from the water column. The longer jaws of pomacentrid planktivores increase the length of the outlever during jaw opening and closing (Figures 3 and 4), a configuration that lends itself to the production of fast mouth movements, and both the A1 and A2 biting muscles tend to have low MA values (Additional files 2 and 4), which further contribute to the generation of fast bites.

Compared to non-planktivorous damselfishes, pomacentrid planktivores can protrude their upper jaws greater distances with higher efficiency of movement (Figures 3, 4, 5; Additional files 3 and 4). The longer ascending processes on the premaxillae of planktivores (Figures 3 and 4) allows for a greater degree of jaw protrusion, an action that rapidly closes the distance between a fish and its prey during a feeding strike. The lower jaws of perciform fishes are indirectly linked to their premaxillae by means of a movable, toothless maxilla that does not form part of the bite [2,30,41]. When the lower jaw is abducted, the maxilla transfers this motion to the premaxilla in such a way that it is protruded, and the protrusion distance that can be achieved without the premaxilla becoming disassociated from the neurocranium is determined by the length of its ascending process. The importance of premaxillary protrusion for damselfish planktivory is supported by the efficiency with which they produce this movement (Additional files 3 and 4). Nearly all of the planktivorous species examined had high protrusion KT scores (Additional file 3), and this was the only KT parameter where the mean scores of individual species distinguished between damselfish tropic groups (Additional files 4 and 5).

Although herbivores are morphologically and biomechanically different from most planktivores, the differences between herbivores and omnivores are far less pronounced. Only the mechanical advantages employed by their A2 muscles significantly distinguish these two groups (Additional file 4), and there is a high degree of overlap in the types of head morphologies that they possess (Figure 3). The overlapping shape and functional distributions of herbivores and omnivores may indicate that there are certain requirements for damselfishes that take in benthic algae as an important component of their diet, regardless of whether or not they are highly herbivorous. Any algae that is consumed must be bitten through or scraped from the substrate, and the production of some minimal amount of force will be required in order to accomplish this.

### Trophic evolution and mechanical advantage

The possession of multiple jaw closing muscles allows for the possibility of having some insertion points that are well situated for efficiently transferring force, and others that generate speed. Such arrangements could permit the exploitation of a broader diet, or allow for one *AM *division to become highly specialized for either speed or force, while other divisions maintain a necessary force/speed balance during biting. That individual species exhibit strong differences in the comparative rank of the MA scores associated with their three biting muscles (Additional file 2) reinforces arguments that the structural division of jaw muscles may allow for functional diversification [28,42-44]. This possibility is strongly supported by the observations that the evolution of the mechanical advantages employed by the A1, A2, and A3 have not been significantly correlated during the damselfish radiation (Figure 5), and that none have experienced significant levels of phylogenetic constraint (Additional file 6). The lack of correlated evolution among the MA values employed by these muscles has implications for evolutionary-developmental studies of the functional anatomy of fish feeding, since recent work suggests that independently functioning divisions of fish biting muscles can develop from a single larval jaw adductor [45]. Our preliminary inspections of larval damselfish skulls (unpublished) also indicate that pomacentrid jaw adductors develop from a single muscle.

The bite force exerted by a muscle is determined by both its mass and the mechanical advantage it employs during contraction. We have demonstrated that the evolution of the simple mechanical advantages employed by the three damselfish biting muscles have not been correlated, but Hulsey et al. [46] determined that the evolution of the muscle masses of these same elements has been highly correlated in the cichlid fishes (Perciformes, Cichlidae) of Lake Malawi in East Africa, a lineage that is closely related to the damselfishes [47-49]. These studies have examined different components of the same biting mechanism in two different fish clades of markedly different ages [the Lake Malawi cichlid radiation is only 1–2 my old; [50]]. Whether or not the evolution of the bite forces generated by the different *AM *muscles of these lineages have experienced correlated evolution within either clade is unknown, and invites further investigation.

Jaw opening MA values are generally low and they have the narrowest range compared to the other MA parameters calculated (Additional file 2). The scores for this parameter do, however, show some ability to differentiate between trophic groups (Additional file 4). The correlation between lower jaw length and CV 1 and RW1 (Figures 3 and 4), the fact that herbivores and planktivores tend to cluster separately on this axis, and the significantly different jaw opening MA scores of these two groups (Additional file 4), support the conclusion that the trophic differences associated with changes in jaw opening MA values are caused primarily by variation in lower jaw length (outlever), as opposed to differences in the size of the angular process of the lower jaw (the jaw opening inlever).

### Trophic evolution and kinematic transmission

Damselfish KT values exhibited a higher frequency of correlated evolution than did their MA values (Figure 5), and except for protrusion KT, a lower connection with damselfish trophic evolution than most of the MA values (Additional file 4). That protrusion KT was determined to be an important, and quickly evolving (low level of phylogenetic signal), aspect of damselfish jaw kinesis supports the idea that that the advantages conferred by the development of highly kinetic jaws are chiefly embodied within the utility of having protrusible premaxillae. Functional variability in the efficiency with which motion is transmitted through the jaw linkages of damselfishes may be predominantly directed at jaw protrusion, whereas selection may be able to individually target the MA parameters examined here due to a higher level of evolutionary independence.

Previous descriptions and simulations of jaw mechanics in wrasses (Perciformes, Labridae) have demonstrated that specific maxillary KT values may be associated with a range of fish jaw morphologies [14,20,51]. This is seen in our damselfish data as well (Figure 3 and Additional file 3), but in contrast to previous work with labrids [5,52], we do not find that maxillary KT values are significantly correlated with trophic ecology in pomacentrids (Additional files 3 and 4). The fact that the same maxillary KT value can be associated with multiple morphologies has been interpreted as support for the idea that morphological diversity may not always be an accurate predictor of biomechanical diversity, or in other words, that there may be a "many-to-one" relationship between form and function [14,51,53-55]. We disagree with this approach and do not interpret our results as support for this concept. Maxillary rotation in the perciform jaw linkage contributes to mouth opening and produces upper jaw protrusion, yet the timing, direction, force and speed of these actions are also determined by many additional anatomical features, and the use of KT ratios to determine functional equivalence has a number of important problems.

One issue with examining convergence (a more traditional name for many-to-one-mapping) using KT values is the fact that the variable is a ratio, and so by definition will assume a similar value with multiple (indeed infinite) combinations of numerator and denominator. So for KT ratio, a many to one relationship is part of the definition of the variable. It is important to recognize that the use of ratios can result in analytical issues such as spurious correlation [51-56], and that such concerns might also apply to our analyses of kinematic parameters due to the fact that the lower jaw rotation angle and lower jaw length are the denominators in all KT and MA calculations, respectively. However, if the use of KT and MA ratios are used as heuristics of feeding performance, rather than assessing more complex biomechanical trends such as functional equivalence, then we suggest that their use is justified.

The recent application of many-to-one mapping using four-bar linkages extrapolates biomechanical similarity between disparate organisms, but does this by focusing on only a small part of the integrated and complex system of movements and structures that are employed during perciform fish feeding. We suggest that this is not appropriate, as similar maxillary KT values do not confer functional equivalence between different jaw morphologies, because differences in jaw adductor anatomy, tooth shape, premaxillary structure, and overall body size, to name but a few features, combine with particular maxillary KT values so as to produce the trophic morphology and behaviour of a perciform fish. The many-to-one concept may have something to offer students of functional morphology, but we suggest that it can only be usefully applied to integrated systems as a whole, and that its application to isolated functional components such as a KT ratio of a linkage system has limited utility in evolutionary studies of biomechanics.

The significant association of high protrusion KT values with planktivory (Additional files 3 and 4), and the clear connection between those planktivores with the highest values for this parameter and a particular type of skull morphology that has evolved independently multiple times (*A. hirundo*, *C. punctipinnis*, *L. tapeinosoma*, *P. obtusirostris*, and *T. jordani*; Additional file 3, Figures 2 and 3), suggest that certain mechanisms for transmitting motion through jaw linkages may require, or at least tend to employ, particular anatomical configurations. Our data indicate that there is a tight and biomechanically defined link between structure and the functional ecology of fish jaws, and that maxillary KT plays an important but not uniquely determinant role in this association. More detailed structural and functional analyses of the primary kinematic variables involved in fish biting (e.g., jaw bone rotations, the production of gape and upper jaw protrusion), combined with the heuristic predictions of speed and motion tracking that modelling provides, and direct performance measures such as kinematic feeding studies, are required to assess levels of convergence and phylogenetic independence among these key traits across a wide range of taxa.

### Jaw kinesis and trophic diversification

Changes in the biomechanics of the maxilla and premaxilla have been a major component of the trophic diversification of the Pomacentridae. The A1 muscle retracts the maxilla, and the MA values associated with its insertion point have the largest maximum value and the greatest range of values of the three biting muscles (Additional file 2). The movement of the maxilla controls the protrusion or retraction of the upper jaw via a ligamentous connection to the dentigerous arm of the premaxilla, and the efficiency of premaxillary protrusion was the only kinetic parameter that distinguished among damselfish trophic groups. Differences in the length of the ascending arm of the premaxilla (i.e., the potential for upper jaw protrusion) are also associated with CV1 and RW1, the axes significantly distinguish between trophic groups and explain the greatest amount of pomacentrid skull shape variation and which (Figures 3 and 4).

This important association of maxillary and premaxillary kinesis with the functional and morphological diversity of the Pomacentridae reflects a much broader pattern in the evolution of fish feeding. The highly kinetic linkages within the jaws of many fishes are derived from much more static anatomical configurations. The maxilla and premaxilla were formerly fused within an upper jaw that moved as a single unit, and which was not protrusible [56,57]. Instead of acting as a linkage that transmits motion from the lower jaw to the premaxilla, the maxilla formerly participated directly in biting. This continues to be its function within the skulls of many osteichthyans, including the tetrapods and a large number of fish lineages, especially those that are most basal [58]. A more mobile maxilla whose rotation plays an important role in jaw protrusion has arisen more than once among the ancestors of successful teleost fish groups [59]. These include fishes within two very successful lineages: the extremely large and loosely defined Perciformes (the perch-like fishes), and the Cypriniformes [carps and minnows; [59]].

Both of these radiations display extraordinary levels of diversity, incorporating several thousand species, and they have both undergone massive radiations in trophic ecology [43,58-61], a portion of which is seen within the perciform damselfishes. Morphological changes have produced an important degree of diversification in the biomechanics of damselfish maxillae and premaxillae, and these alterations constitute a major component of the biomechanical changes that are clearly associated with differences in their trophic ecology (Additional files 4 and 5). Morphological and biomechanical studies of another fish group, the Labridae (wrasses), have demonstrated that the biomechanics of the maxilla and premaxilla have also been of great importance during the extensive trophic radiation of this diverse lineage [4,5]. It seems probable that the expanding body of work focused on the evolution of fish jaw biomechanics will determine that changes in upper jaw kinesis will have strongly influenced the success of many fish lineages that possess highly mobile jaws.

### The damselfishes as an example of a reticulate adaptive radiation

Although the evolution of multiple aspects of damselfish jaw biomechanics has progressed under low levels of constraint, most of the members of this lineage occupy one of only three major feeding niches [Additional file 1; [10]]. Almost all damselfishes have been classified as either herbivores, planktivores, or omnivores that consume both filamentous algae and small animal prey that are not highly elusive. Examples of the prey taken by omnivorous damselfishes include fish eggs (both demersal and planktonic), zooplankton (including fish and crustacean larvae), and small invertebrates such as polycheate worms, amphipods, hydroids, anthozoans (their polyps and tentacles), tunicates, and the occasional fish ectoparasite [10,62-65]. There are a small number of exceptions to this general pattern. *Cheiloprion labiatus *and *Plectroglyphidodon johnstonianus *feed almost exclusively upon scleractinian coral polyps, although they do not excavate the skeletons [62], *Abudefduf septemfasciatus *and *Nexilosus latifrons *will both take in some degree of hard shelled prey [63,66], and some highly territorial species that have been described as herbivores have been shown to take in large amounts of detritus [67]. These known exceptions account for < 2.0% of the damselfish diversity [10]. There are no piscivorous damselfishes, nor do they specialize on any other elusive prey larger than zooplankton. There are no explicitly durophagous pomacentrids, no adult damselfishes feed extensively on fish ectoparasites, and none are known to nip scales, fins, mucus or pieces of flesh from other animals. Damselfishes rarely feed upon organisms that require any subjugation, and they have a very limited capacity to puncture or crush their prey.

Blomberg et al. [36] suggested that the presence of a low level of phylogenetic signal among the characters of a lineage may indicate an adaptive radiation, but if this is true in the case of the damselfishes, then we have a relatively old (at least 50 my) adaptive radiation whose crown group has progressed in only three primary ecological directions. The solution to this apparent contradiction lies in examining how frequently damselfishes switch trophic habits. Character mapping indicates that all three of the major pomacentrid trophic groups contain members of several different damselfish lineages that have evolved these habits independently [68,69]. Among the Pomacentridae, herbivory and omnivory have both arisen seven times, planktivory four times, and feeding on scleractinian coral polyps twice [68,69]. Part of this pattern can be seen in Figure 2, although this schematic shows only a portion of the 104 species examined by Cooper [68,69].

We find that damselfishes are readily able to evolve their head morphology and jaw biomechanics so that they can shift their feeding habits between herbivory, planktivory, and a limited type of omnivory, but that they have demonstrated little ability to invade other feeding niches. Questions regarding the evolution of damselfish skull biomechanics must therefore be directed at two different aspects of this process: rate and direction. Our evidence indicates that the damselfishes have experienced a low level of constraint on the speed with which they have repeatedly converged on only three primary ecological states.

Have the damselfishes undergone an adaptive radiation? This term is typically used to refer to a lineage that has diverged so as to produce descendant species that occupy a wide variety of ecological niches [70,71]. Classic examples of these amongst the vertebrates would include the cichlid fishes of the East African rift lakes [50], Darwin's finches in the Galapagos Islands [72], and the *Anolis *lizards of the Caribbean [73-75].

The Caribbean *Anolis *lineage, whose more than 300 species have repeatedly evolved only 4 primary ecotypes over 30–40 million years [73-75], represent a case where repeated evolutionary convergence on a limited number of trophic states has been considered to represent an adaptive radiation despite limited ecological divergence. There appear to be two different classes of adaptive radiations, the classic example, where morphological divergence at speciation has produced a wide range of anatomical and ecological diversity (e.g., Darwin's finches, East African rift lake cichlids), and cases in which morphological divergence at speciation has been associated with the repeated convergence on a limited number of ecotypes (e.g., Caribbean *Anolis*, damselfishes). We apply the term *reticulate adaptive radiation *to describe the second class, and use it to refer to lineages whose evolutionary patterns are characterized by rapid and repeated shifts between a limited number of eco-morphological states. Determination of the factors that cause a rapidly evolving and successful lineage to produce a reticulate pattern of adaptive radiation, as opposed to a steadily increasing their eco-morphological diversity, clearly invites further study.

## Conclusion

The use of a single set of homologous coordinates to analyze both the anatomical and mechanical aspects of the functional morphology of damselfish feeding provided two useful and reciprocally illuminating arrays of information. The results of both types of analysis indicate that the morphological and biomechanical adaptations associated with damselfish planktivory represent a major component of their trophic diversification. Whereas plankton is estimated to form a large percentage of the diets of approximately one third of all pomacentrid species [10], the description of planktivorous damselfish head anatomy and biomechanics is of strong importance for understanding the ecology of both the Pomacentridae and the reefs that they occupy.

The independent evolution of the MA parameters associated with the different damselfish biting muscles, the finding that, in several damselfish species, these separate muscles employ very different MAs during jaw adduction, and the pattern of rapid divergence that is pervasive in history of damselfish trophic evolution, all suggest that the division of the single, ancestral *adductor mandibulae *has promoted functional diversification. Changes in jaw kinesis have likewise been of fundamental importance to the evolution of damselfish feeding, particularly those changes that are associated with the efficiency of jaw protrusion. We found a tight correspondence between skull morphology and jaw biomechanics, and no support for functional equivalence between multiple anatomical configurations among the Pomacentridae. It is our hope that further evolutionary studies of perciform bite mechanics will rigorously test the alternative hypotheses of "many-to-one mapping" and the functional uniqueness of different skull morphologies.

The trophic evolution of the damselfishes is characterized by rapid and repeated shifts between a small number of trophic niches, and we use the term reticulate adaptive radiation to describe this pattern. We suggest that the rapid eco-morphological evolution of a lineage need not be coupled with a rapid expansion into an increasingly greater number of niches. It is our belief that investigation into the factors that either link or decouple the rapid evolution of eco-morphological characters with/from the ecological expansion of a clade will be a productive area of future investigation.

The Pomacentridae represent only a small component of the tremendous perciform radiation, an incredibly diverse lineage of roughly twelve thousand species that dominates many of the worlds aquatic systems, and which represents one of the most successful branches of the vertebrata. The accumulation of knowledge about the functional morphology of feeding within this group has seen a dramatic increase in recent years [e.g., [4,5,12,13,20,52,76-84]], and broad patterns of evolution are beginning to emerge. Further work will allow us to determine which modes of evolution have been prevalent during the expansion of the Perciformes. Phylogenetic comparative analyses of morphological and biomechanical data constitute a powerful approach to studying evolution, and future endeavours that combine these techniques show great promise for determining the means by which specific anatomical changes have generated ecological diversity via functional divergence.

## Authors' contributions

JC and MW both contributed to the collection of pomacentrid specimens from the field. JC performed the dissections, photography and morphometric analyses. MW created the software program Jawsmodel4, and used it to perform the biomechanical analyses of damselfish skulls. JC performed the statistical and phylogenetic comparative analyses of the biomechanical data generated by Jawsmodel4. JC conceived of the study, and was guided during its execution by MW. JC created the initial drafts of the manuscript, its figures and tables. Both authors read and approved the final manuscript.

## Supplementary Material

Additional file 1 **Trophic classifications of the fishes examined.** Trophic classifications of the damselfish species examined, with museum specimen identification information Click here for file

Additional file 2 **Damselfish species ranked by mean MA values.** Damselfish species ranked by mean MA values. Trophic categories are indicated by colour codes. Click here for file

Additional file 3 **Damselfish species ranked by mean KT values.** Damselfish species ranked by mean KT values. Trophic categories are indicated by colour codes Click here for file

Additional file 4 **Pairwise ANOVA results for comparisons of the biomechanics of damselfish trophic groups.** Results of ANOVA tests Click here for file

Additional file 5 **Selection summary for a stepwise DFA of parameters that discriminate between major damselfish trophic groups.** DFA results Click here for file

Additional file 6 **Results of randomization and linear regression model tests for phylogenetic signal.** Results of phylogenetic tests Click here for file
