# Supplementary material for: Form and function of damselfish skulls: rapid and repeated evolution into a limited number of trophic niches
Source: BMC Evol Biol. 2009 Jan 30;9:24. doi: 10.1186/1471-2148-9-24 (PMC2654721; doi:10.1186/1471-2148-9-24)
Supplement: Additional file 1 — Trophic classifications of the fishes examined. Trophic classifications of the damselfish species examined, with museum specimen identification information [file 1471-2148-9-24-S1.doc]

Trophic classifications of the fishes examined. Planktivores (red), Herbivores (green), Omnivores (blue), AMS = Australian museum, FMNH = Field museum, SIO = Scripps Institution of Oceanography, USNM = U.S. National Museum of Natural History

Number of specimens Species Tropic habit citation Specimen numbers

3 ***Abudefduf vaigiensis*** Sano et al., 1984 FMNH uncataloged

2 ***Acanthochromis polyacanthus*** Westneat and Resing, 1988 FMNH: 23749 & 23752

2 ***Altrichthys curatus*** (juveniles only) Allen, 1999 USNM 282339

3 ***Amblyglyphidodon curacao*** Sano et al., 1984 FMNH uncataloged

3 ***Amblypomacentrus clarus*** Allen, 1991 FMNH uncataloged

(inferred from diet data for *A. breviceps*)

3 ***Amphiprion akindynos*** Sano et al., 1984 FMNH uncataloged

3 ***Cheiloprion labiatus*** (Coral polyps) Sano et al., 1984 FMNH 51983

3 ***Chromis amboinensis*** Allen, 1991 FMNH uncataloged

3 ***Chromis hirundo* (*Azurina hirundo*)** Allen, 1991 SIO 54-219

3 ***Chromis punctipinnis*** Allen, 1991 SIO H53-103

3 ***Chrysiptera cyanea*** Sano et al., 1984 FMNH uncataloged

3 ***Dascyllus melanurus*** Allen, 1991 FMNH uncataloged

1 ***Dischistodus melanotus*** Allen, 1991 FMNH uncataloged

3 ***Hemiglyphidodon plagiometopon*** Allen, 1991 FMNH 47850

3 ***Hypsypops rubicundus*** Allen, 1991 SIO 60-14

3 ***Lepidozygus tapeinosoma*** Allen, 1991 AMS I.37334-004

3 ***Mecaenichthys immaculatus*** Allen, 1991 AMS: B.7221 & I.19103-030

3 ***Microspathodon dorsalis*** Cooper, 2006 FMNH 74800

3 ***Neoglyphidodon nigroris*** Allen, 1991 FMNH uncataloged

3 ***Neopomacentrus azysron*** Allen, 1991 FMNH 110620

1 ***Nexilosus latifrons*** Allen, 1991 FMNH 74806

3 ***Parma microlepis*** Allen, 1991 FMNH: 61889 &113689

3 ***Plectroglyphidodon lacrymatus*** Sano et al., 1984 FMNH 112502

3 ***Pomachromis richardsoni*** Sano et al., 1984 AMS: I.16641-008, I.16504-010, I.17470-008

3 ***Pomacentrus alexanderae*** Allen, 1991 FMNH uncataloged

2 ***Premnas biaculeatus*** Allen, 1991 FMNH uncataloged

3 ***Pristotis obtusirostris*** G. R. Allen, pers. comm. AMS I.15557-199

3 ***Similiparma hermani*** Allen, 1991 FMNH uncataloged

3 ***Stegastes flavilatus*** Allen, 1991 FMNH 61842

3 ***Teixeirichthys jordani*** G. R. Allen, pers. comm. USNM 306140
