# Supplementary material for: Form and function of damselfish skulls: rapid and repeated evolution into a limited number of trophic niches
Source: BMC Evol Biol. 2009 Jan 30;9:24. doi: 10.1186/1471-2148-9-24 (PMC2654721; doi:10.1186/1471-2148-9-24)
Supplement: Additional file 2 — Damselfish species ranked by mean MA values. Damselfish species ranked by mean MA values. Trophic categories are indicated by colour codes. [file 1471-2148-9-24-S2.doc]

Damselfish species ranked by mean MA values. Jaw opening MA=JOMA. Planktivores (red), Herbivores (green), Omnivores (blue)

| **Species** | **JO**  **MA** | **Species** | **A1**  **MA** | **Species** | **A2**  **MA** | **Species** | **A3**  **MA** |
| --- | --- | --- | --- | --- | --- | --- | --- |
| ***Chrysiptera cyanea*** | **0.12** | ***Chromis hirundo*** | **0.24** | ***Lepidozygus tapeinosoma*** | **0.25** | ***Neoglyphidodon nigroris*** | **0.22** |
| ***Chromis punctipinnis*** | **0.14** | ***Pristotis obtusirostris*** | **0.33** | ***Teixeirichthys jordani*** | **0.32** | ***Amphiprion akindynos*** | **0.23** |
| ***Stegastes flavilatus*** | **0.15** | ***Chromis punctipinnis*** | **0.35** | ***Microspathodon dorsalis*** | **0.36** | ***Neopomacentrus azysron*** | **0.23** |
| ***Chromis amboinensis*** | **0.16** | ***Dascyllus melanurus*** | **0.37** | ***Pristotis obtusirostris*** | **0.36** | ***Similiparma hermani*** | **0.23** |
| ***Chromis hirundo*** | **0.16** | ***Neopomacentrus azysron*** | **0.63** | ***Chromis punctipinnis*** | **0.38** | ***Chromis amboinensis*** | **0.26** |
| ***Neopomacentrus azysron*** | **0.17** | ***Chromis amboinensis*** | **0.64** | ***Chrysiptera cyanea*** | **0.40** | ***Amblypomacentrus clarus*** | **0.27** |
| ***Mecaenichthys immaculatus*** | **0.18** | ***Teixeirichthys jordani*** | **0.67** | ***Neopomacentrus azysron*** | **0.40** | ***Abudefduf vagiensis*** | **0.29** |
| ***Pomacentrus alexanderae*** | **0.18** | ***Amblypomacentrus clarus*** | **0.69** | ***Hemiglyphidodon plagiometopon*** | **0.40** | ***Stegastes flavilatus*** | **0.29** |
| ***Pristotis obtusirostris*** | **0.18** | ***Pomachromis richardsoni*** | **0.77** | ***Amblypomacentrus clarus*** | **0.41** | ***Chrysiptera cyanea*** | **0.29** |
| ***Acanthochromis polyacanthus*** | **0.19** | ***Lepidozygus tapeinosoma*** | **0.84** | ***Mecaenichthys immaculatus*** | **0.42** | ***Hypsypops rubicundus*** | **0.29** |
| ***Amblyglyphidodon curacao*** | **0.19** | ***Premnas biaculeatus*** | **0.88** | ***Neoglyphidodon nigroris*** | **0.43** | ***Premnas biaculeatus*** | **0.30** |
| ***Pomachromis richardsoni*** | **0.19** | ***Similiparma hermani*** | **0.91** | ***Chromis hirundo*** | **0.44** | ***Microspathodon dorsalis*** | **0.31** |
| ***Amphiprion akindynos*** | **0.19** | ***Amblyglyphidodon curacao*** | **1.00** | ***Dascyllus melanurus*** | **0.45** | ***Pomachromis richardsoni*** | **0.32** |
| ***Teixeirichthys jordani*** | **0.20** | ***Nexilosus latifrons*** | **1.13** | ***Pomachromis richardsoni*** | **0.47** | ***Chromis punctipinnis*** | **0.34** |
| ***Hypsypops rubicundus*** | **0.20** | ***Cheiloprion labiatus*** | **1.20** | ***Acanthochromis polyacanthus*** | **0.47** | ***Pristotis obtusirostris*** | **0.34** |
| ***Dascyllus melanurus*** | **0.21** | ***Chrysiptera cyanea*** | **1.21** | ***Chromis amboinensis*** | **0.50** | ***Cheiloprion labiatus*** | **0.35** |
| ***Premnas biaculeatus*** | **0.21** | ***Amphiprion akindynos*** | **1.25** | ***Amphiprion akindynos*** | **0.50** | ***Plectroglyphidodon lacrymatus*** | **0.36** |
| ***Amblypomacentrus clarus*** | **0.22** | ***Microspathodon dorsalis*** | **1.39** | ***Stegastes flavilatus*** | **0.51** | ***Mecaenichthys immaculatus*** | **0.36** |
| ***Abudefduf vagiensis*** | **0.23** | ***Stegastes flavilatus*** | **1.48** | ***Dischistodus melanotus*** | **0.51** | ***Dischistodus melanotus*** | **0.37** |
| ***Plectroglyphidodon lacrymatus*** | **0.23** | ***Acanthochromis polyacanthus*** | **1.50** | ***Parma microlepis*** | **0.52** | ***Parma microlepis*** | **0.37** |
| ***Lepidozygus tapeinosoma*** | **0.23** | ***Hypsypops rubicundus*** | **1.52** | ***Nexilosus latifrons*** | **0.52** | ***Acanthochromis polyacanthus*** | **0.38** |
| ***Nexilosus latifrons*** | **0.23** | ***Neoglyphidodon nigroris*** | **1.85** | ***Pomacentrus alexanderae*** | **0.52** | ***Pomacentrus alexanderae*** | **0.39** |
| ***Dischistodus melanotus*** | **0.24** | ***Dischistodus melanotus*** | **1.91** | ***Amblyglyphidodon curacao*** | **0.53** | ***Hemiglyphidodon plagiometopon*** | **0.41** |
| ***Similiparma hermani*** | **0.25** | ***Parma microlepis*** | **1.95** | ***Cheiloprion labiatus*** | **0.53** | ***Lepidozygus tapeinosoma*** | **0.41** |
| ***Neoglyphidodon nigroris*** | **0.28** | ***Plectroglyphidodon lacrymatus*** | **2.18** | ***Plectroglyphidodon lacrymatus*** | **0.53** | ***Nexilosus latifrons*** | **0.41** |
| ***Hemiglyphidodon plagiometopon*** | **0.30** | ***Pomacentrus alexanderae*** | **2.41** | ***Premnas biaculeatus*** | **0.53** | ***Dascyllus melanurus*** | **0.44** |
| ***Parma microlepis*** | **0.30** | ***Hemiglyphidodon plagiometopon*** | **2.47** | ***Hypsypops rubicundus*** | **0.56** | ***Teixeirichthys jordani*** | **0.44** |
| ***Cheiloprion labiatus*** | **0.32** | ***Mecaenichthys immaculatus*** | **2.62** | ***Abudefduf vagiensis*** | **0.59** | ***Amblyglyphidodon curacao*** | **0.47** |
| ***Microspathodon dorsalis*** | **0.33** | ***Abudefduf vagiensis*** | **2.95** | ***Similiparma hermani*** | **0.64** | ***Chromis hirundo*** | **0.48** |
