# Supplementary material for: Form and function of damselfish skulls: rapid and repeated evolution into a limited number of trophic niches
Source: BMC Evol Biol. 2009 Jan 30;9:24. doi: 10.1186/1471-2148-9-24 (PMC2654721; doi:10.1186/1471-2148-9-24)
Supplement: Additional file 3 — Damselfish species ranked by mean KT values. Damselfish species ranked by mean KT values. Trophic categories are indicated by colour codes [file 1471-2148-9-24-S3.doc]

Damselfish species ranked by mean KT values. Maxillary KT=MKT. Gape KT=GKT. Protrusion KT=PKT.

Planktivores (red), Herbivores (green), Omnivores (blue)

| **Species** | **M**  **KT** | **Species** | **G**  **KT** | **Species** | **P**  **KT** |
| --- | --- | --- | --- | --- | --- |
| ***Microspathodon dorsalis*** | **0.60** | ***Teixeirichthys jordani*** | **0.58** | ***Microspathodon dorsalis*** | **0.38** |
| ***Chromis punctipinnis*** | **0.88** | ***Similiparma hermani*** | **0.82** | ***Premnas biaculeatus*** | **0.42** |
| ***Mecaenichthys immaculatus*** | **0.88** | ***Chromis punctipinnis*** | **0.82** | ***Neopomacentrus azysron*** | **0.42** |
| ***Amblypomacentrus clarus*** | **0.92** | ***Amblypomacentrus clarus*** | **0.83** | ***Amphiprion akindynos*** | **0.44** |
| ***Pristotis obtusirostris*** | **0.95** | ***Microspathodon dorsalis*** | **0.83** | ***Similiparma hermani*** | **0.45** |
| ***Neoglyphidodon nigroris*** | **1.00** | ***Pristotis obtusirostris*** | **0.85** | ***Mecaenichthys immaculatus*** | **0.45** |
| ***Similiparma hermani*** | **1.01** | ***Dischistodus melanotus*** | **0.91** | ***Chrysiptera cyanea*** | **0.48** |
| ***Acanthochromis polyacanthus*** | **1.06** | ***Hypsypops rubicundus*** | **0.99** | ***Acanthochromis polyacanthus*** | **0.52** |
| ***Parma microlepis*** | **1.06** | ***Cheiloprion labiatus*** | **1.04** | ***Neoglyphidodon nigroris*** | **0.53** |
| ***Premnas biaculeatus*** | **1.08** | ***Nexilosus latifrons*** | **1.16** | ***Chromis amboinensis*** | **0.53** |
| ***Hemiglyphidodon plagiometopon*** | **1.09** | ***Plectroglyphidodon lacrymatus*** | **1.20** | ***Hemiglyphidodon plagiometopon*** | **0.54** |
| ***Neopomacentrus azysron*** | **1.11** | ***Chromis hirundo*** | **1.21** | ***Plectroglyphidodon lacrymatus*** | **0.56** |
| ***Chrysiptera cyanea*** | **1.11** | ***Premnas biaculeatus*** | **1.21** | ***Stegastes flavilatus*** | **0.56** |
| ***Hypsypops rubicundus*** | **1.11** | ***Amblyglyphidodon curacao*** | **1.22** | ***Pomacentrus alexanderae*** | **0.57** |
| ***Lepidozygus tapeinosoma*** | **1.15** | ***Parma microlepis*** | **1.23** | ***Hypsypops rubicundus*** | **0.57** |
| ***Nexilosus latifrons*** | **1.17** | ***Neopomacentrus azysron*** | **1.23** | ***Abudefduf vagiensis*** | **0.57** |
| ***Cheiloprion labiatus*** | **1.18** | ***Acanthochromis polyacanthus*** | **1.24** | ***Parma microlepis*** | **0.57** |
| ***Amblyglyphidodon curacao*** | **1.25** | ***Neoglyphidodon nigroris*** | **1.25** | ***Pomachromis richardsoni*** | **0.57** |
| ***Stegastes flavilatus*** | **1.26** | ***Mecaenichthys immaculatus*** | **1.26** | ***Dascyllus melanurus*** | **0.58** |
| ***Dischistodus melanotus*** | **1.26** | ***Stegastes flavilatus*** | **1.27** | ***Cheiloprion labiatus*** | **0.59** |
| ***Amphiprion akindynos*** | **1.27** | ***Lepidozygus tapeinosoma*** | **1.28** | ***Amblypomacentrus clarus*** | **0.61** |
| ***Dascyllus melanurus*** | **1.29** | ***Dascyllus melanurus*** | **1.31** | ***Lepidozygus tapeinosoma*** | **0.62** |
| ***Plectroglyphidodon lacrymatus*** | **1.29** | ***Abudefduf vagiensis*** | **1.34** | ***Nexilosus latifrons*** | **0.64** |
| ***Pomachromis richardsoni*** | **1.31** | ***Chrysiptera cyanea*** | **1.38** | ***Pristotis obtusirostris*** | **0.64** |
| ***Pomacentrus alexanderae*** | **1.33** | ***Pomachromis richardsoni*** | **1.41** | ***Amblyglyphidodon curacao*** | **0.65** |
| ***Abudefduf vagiensis*** | **1.40** | ***Amphiprion akindynos*** | **1.41** | ***Chromis punctipinnis*** | **0.74** |
| ***Chromis amboinensis*** | **1.48** | ***Hemiglyphidodon plagiometopon*** | **1.41** | ***Dischistodus melanotus*** | **0.77** |
| ***Teixeirichthys jordani*** | **1.49** | ***Chromis amboinensis*** | **1.41** | ***Chromis hirundo*** | **0.95** |
| ***Chromis hirundo*** | **1.93** | ***Pomacentrus alexanderae*** | **1.42** | ***Teixeirichthys jordani*** | **1.11** |
