# Supplementary material for: Form and function of damselfish skulls: rapid and repeated evolution into a limited number of trophic niches
Source: BMC Evol Biol. 2009 Jan 30;9:24. doi: 10.1186/1471-2148-9-24 (PMC2654721; doi:10.1186/1471-2148-9-24)
Supplement: Additional file 4 — Pairwise ANOVA results for comparisons of the biomechanics of damselfish trophic groups. Results of ANOVA tests [file 1471-2148-9-24-S4.doc]

Pairwise ANOVA results for comparisons of the biomechanics of damselfish trophic groups (d.f.=2). The results of all tests for the significance of individual comparisons concurred.

Jaw opening MA=JOMA. Maxillary KT=MKT. Gape KT=GKT. Protrusion KT=PKT.

| Parameter | F Value | p-value | significant pairwise comparisons |  | Parameter | F Value | p-value | significant pairwise comparison |
| --- | --- | --- | --- | --- | --- | --- | --- | --- |
| JOMA | 3.390 | 0.039 | Herb. > Plank. |  | A3MA | 1.720 | 0.185 |  |
| A1MA | 9.520 | <0.001 | Herb. > Plank. |  | MKT | 0.740 | 0.480 |  |
|  |  |  | Omn. > Plank. |  | GKT | 4.410 | 0.190 |  |
| A2MA | 18.240 | <0.001 | Omn. > Plank. |  | PKT | 11.060 | <0.001 | Plank. > Herb. |
|  |  |  | Omn. > Herb. |  |  |  |  | Plank. > Omn. |
