# Supplementary material for: Form and function of damselfish skulls: rapid and repeated evolution into a limited number of trophic niches
Source: BMC Evol Biol. 2009 Jan 30;9:24. doi: 10.1186/1471-2148-9-24 (PMC2654721; doi:10.1186/1471-2148-9-24)
Supplement: Additional file 5 — Selection summary for a stepwise DFA of parameters that discriminate between major damselfish trophic groups. DFA results [file 1471-2148-9-24-S5.doc]

Selection summary for a stepwise DFA of parameters that discriminate between major damselfish trophic groups.

Protrusion KT=PKT

Variable Partial Wilks' Pr < Average squared Pr >

Step entered R-Square F Value Pr > F Lambda Lambda canonical correlation ASCC

1 A2MA 0.3214 18.24 < 0.0001 0.67857319 < 0.0001 0.16071340 < 0.0001

2 PKT 0.2305 11.38 < 0.0001 0.52216277 < 0.0001 0.24521549 < 0.0001

3 A1MA 0.1405 6.13 0.0034 0.44877667 < 0.0001 0.29205448 < 0.0001
