# Supplementary material for: Form and function of damselfish skulls: rapid and repeated evolution into a limited number of trophic niches
Source: BMC Evol Biol. 2009 Jan 30;9:24. doi: 10.1186/1471-2148-9-24 (PMC2654721; doi:10.1186/1471-2148-9-24)
Supplement: Additional file 6 — Results of randomization and linear regression model tests for phylogenetic signal. Results of phylogenetic tests [file 1471-2148-9-24-S6.doc]

Results of randomization and linear regression model tests for phylogenetic signal.

Jaw opening MA=JOMA. Maxillary KT=MKT. Gape KT=GKT. Protrusion KT=PKT.

biomechanical p-values from PHYSIG ln ML for ln ML for AIC for AIC for

parameter randomization test OLS model PGLS model OLS model PGLS model

JOMA 0.740 51.8 > 49.1 - 95.6 < - 90.2

A1MA 0.053 - 23.2 > - 27.0 54.4 < 61.9

A2MA 0.100 35.4 > 30.6 - 62.8 < - 53.2

A3MA 0.623 35.4 > 30.2 - 62.8 < - 52.5

MKT 0.233 0.7 > - 2.3 6.6 < 12.5

GKT 0.025 2.9 < 3.2 2.2 > 1.6

PKT 0.123 16.5 > 12.7 -25.0 < - 17.3
